# Rickettsiosis with Pleural Effusion: A Systematic Review with a Focus on Rickettsiosis in Italy

**DOI:** 10.3390/tropicalmed7010011

**Published:** 2022-01-14

**Authors:** Cristoforo Guccione, Raffaella Rubino, Claudia Colomba, Antonio Anastasia, Valentina Caputo, Chiara Iaria, Antonio Cascio

**Affiliations:** 1 Department of Health Promotion, Mother and Child Care, Internal Medicine and Medical Specialties, University of Palermo, 90127 Palermo, Italy; cristoforo.guccione@you.unipa.it (C.G.); claudia.colomba@unipa.it (C.C.); antonioanastasia90@gmail.com (A.A.); valentina.caputo@unipa.it (V.C.); 2 Infectious and Tropical Disease Unit, AOU Policlinico “P. Giaccone”, 90127 Palermo, Italy; raffaella.rubino@policlinico.pa.it; 3 Infectious and Tropical Disease Unit—ARNAS Civico, 90127 Palermo, Italy; iaria.chiara@gmail.com

**Keywords:** Mediterranean spotted fever, pleural effusion, Rickettsiales, *Rickettsia*, rickettsiosis, TIBOLA, anaplasmosis, Italy

## Abstract

Background: Motivated by a case finding of Mediterranean spotted fever (MSF) associated with atypical pneumonia and pleural effusion in which *Rickettsia conorii subsp. israelensis* was identified by molecular methods in the pleural fluid, we wanted to summarize the clinical presentations of rickettsiosis in Italy by systematic research and to make a systematic review of all the global cases of rickettsiosis associated with pleural effusion. Methods: For the literature search, the Preferred Reporting Items for Systematic Reviews and Meta-Analyses (PRISMA) methodology was followed. We chose to select only the studies published in last 25 years and confirmed both with serological and molecular assays. Results: Human cases of rickettsiosis in Italy were reported in 48 papers describing 2831 patients with very different clinical presentations; the majority was MSF accounted to *R. conorii* and was reported in Sicily. Pleural effusion associated with infection with microorganisms belonging to Rickettsiales was described in 487 patients. It was rarely associated with microorganisms different from *O. tsutsugamushi;* also rarely, cases of scrub typhus were reported outside Southeast Asia and in the largest majority, the diagnosis was achieved with serology. Conclusions: MSF, especially when caused by *R. conorii subsp. israelensis,* may be a severe disease. A high index of suspicion is required to promptly start life-saving therapy. Pleural effusion and interstitial pneumonia may be part of the clinical picture of severe rickettsial disease and should not lead the physician away from this diagnosis

## 1. Introduction

The most common Italian rickettsiosis is Mediterranean Spotted Fever (MSF). About 400 cases of MSF are reported every year, most of which in people residing in Sicily, Sardinia and Southern Italy with a mortality of less than 3% [1]. MSF is commonly associated with a symptom triad consisting of fever, cutaneous rash, and *tache noire.* The rash is usually maculopapular but, especially in severe presentation, can be petechial. The *tache noire*, translatable from French as “black spot”, is the typical, painless and non-pruritic eschar at the site of the arthropod bite. Other typical findings are arthromyalgia and headache. However, in recent years, other rickettsiosis such as TIBOLA/DEBONEL/SENLAT (Tick-borne lymphadenopathy/dermacentor borne necrosis erythema and lymphadenopathy/scalp eschar and neck lymphadenopathy after tick bite [2]), and many other *Rickettsia* spp. or subspecies have been identified in humans, vector arthropods and animals [2].

Motivated by a case finding of MSF associated with atypical pneumonia and pleural effusion in which *R. conorii subsp. israelensis* was identified by molecular methods in the pleural fluid [3] we wanted to take stock of the clinical presentations of rickettsiosis in Italy by systematic research and to make a systematic review of all the global cases of rickettsiosis associated with pleural effusion. Due to the differences between the features of the two reviews and their purposes we made and reported their results of them separately. 

## 2. Materials and Methods

**Figure 1:**
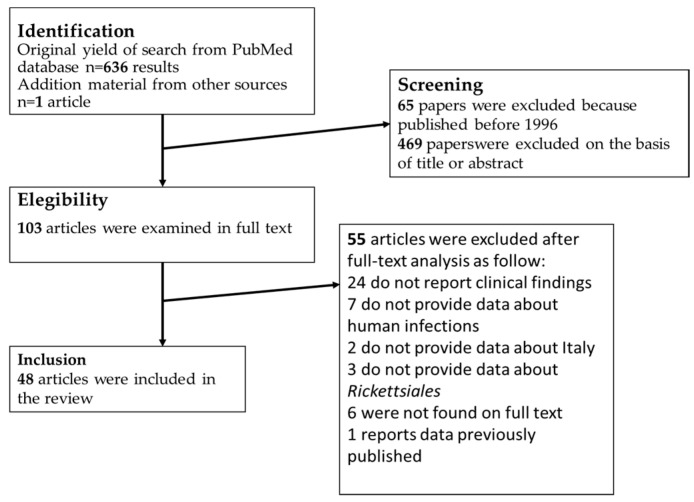
Process of bibliographic research for review about Rickettsiales infections in Italy.

We considered rickettsiosis as all the diseases caused by microorganisms included in the family Rickettsiales. For the literature search, the Preferred Reporting Items for Systematic Reviews and Meta-Analyses (PRISMA) methodology was followed [4]. The terms: “(Rickett* OR Anaplasm* OR Ehrlichi* Orientia) AND Italy)” were associated in PubMed to find papers on rickettsiosis in Italy. The terms “(Ricketts* OR Orientia OR Anaplasm* OR Ehrlichi* OR Scrub Typhus) AND (pleur* OR effusion)” without geographical restrictions, were used to find papers describing cases of rickettsiosis with pleural effusion. All references listed were hand-searched for other relevant articles. For both of the reviews, we chose to include only studies published in the last 25 years, thus from 1996 to 2021, furthermore, studies available only in a language different from Latin and Germanic languages were excluded (Figure 1). All data were recorded and analyzed in Excel sheets. For both of the searches, we included cases in which the diagnosis was made by serological or molecular methods. The term Israeli spotted fever (ISF) was used to identify cases of MSF caused by *R. conorii subsp. israelensis*.

**Figure 2:**
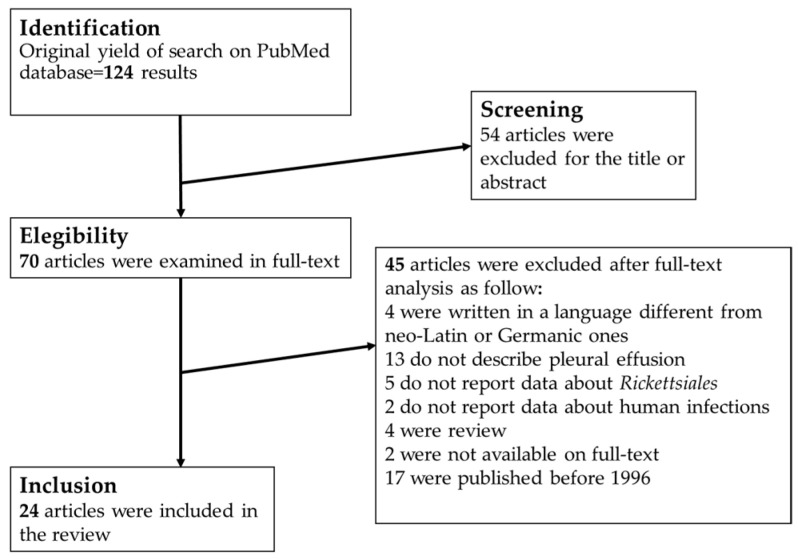
Process of bibliographic research for review about pleural effusion in course of Rickettsiales infection in the world.

The characteristics of each case are analytically showed in Supplementary File. The process for the review of the cases of rickettsiosis associated with pleural effusion is summarized in Figure 2. Ultimately, 25 studies describing 487 human cases of rickettsiosis with pleural effusion were included in this review, and the characteristics of the cases can be found in the Supplementary File. The Supplementary File contains 3 Excel sheets: (1) Rickettsiales infection in Italy; (2) atypical presentation in Italy and (3) pleural effusion in course of infection by Rickettsiales in the world. 

## 3. Results

### 3.1. Infections in Italy

**Figure 3:**
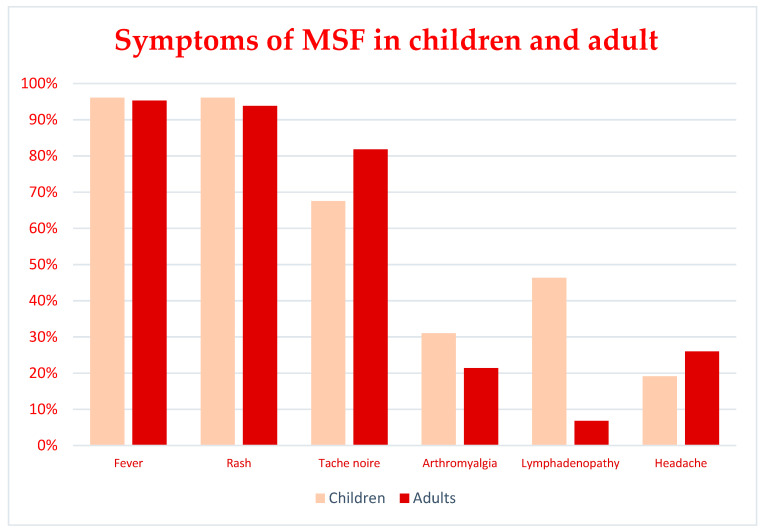
Comparison between main symptoms of MSF in children and adults.

From 1996 to 2021 a total of 2831 cases of rickettsiosis that occurred in Italy were reported in 48 papers. 2787 cases were attributable to MSF [5,6,7,8,9,10,11,12,13,14,15,16,17,18,19,20,21,22,23,24,25,26,27,28,29,30,31,32,33] (only in 56 of the above cases the diagnosis was made by molecular methods), 7 cases of ISF were reported in 2 papers (in both cases the diagnosis was made by molecular methods), [15,34], 9 were Human granulocytic anaplasmosis (HGA) [35,36,37,38] (in 7 of them, the diagnosis was made by molecular methods), 6 were TIBOLA [39,40,41,42] (in all of them, the diagnosis was made by molecular methods), 12 were ATBF [43,44], (in none of them the diagnosis was made by molecular methods), 2 were scrub typhus [45] (one diagnosed with serology and one diagnosed with molecular methods), 1 was a case of murine typhus (serologically diagnosed) [46], one case was ehrlichiosis (serologically diagnosed) [19], and other 7 case reports did present atypical clinical manifestation [47,48,49,50,51]. Almost all the cases came from the major islands. 68.9% of the cases were reported in Sicily (1953 cases) [5,6,7,8,9,10,11,12,13,14,15,20,25,26,31,33,34,36,42], 29.60% in Sardinia (838 cases) [18,19,29,32,35], the remaining 1.4% were reported from the other Italian regions [16,17,21,22,23,24,28,30,38,39,40,41,43,44,46,47,49,50,51]. 18 imported cases were reported [43,44,48,50,51], 12 were African tick bite fevers (ATBF) [43,44,48], 4 were non-specific fever accounted to *R. helvetica* and *R. felis* [50,51]. One was a sacral syndrome in which *R. africae* was identified with molecular techniques [48] and the last two were cases of scrub typhus [45]. Other clinical manifestations included: a case of acute hepatitis caused by *R. aeschlimannii* [47] and a case of thrombosis of the ophthalmic vein accounted to *R. conorii* [49]. 1388 patients with MSF were male (727 children, 129 adults; for 532 the age was not reported) and 817 were female (477 children, 63 adults and, for 287 the age was not reported). In MSF, the mean ages were 47.7 and 5.9 years, respectively for adult and pediatric patients. The characteristics of all the patients with MSF were resumed in Table 1, the demographic and clinical characteristics were reported only for a part of the total of the patients, in Supplementary File the fields for which no information is available were reported with “NR” meaning “Not reported”. In Table 1 is displayed a resume considering only the patients for which these data were available. In the case series of adult patients with MSF, fever was present in 95.3% of patients, rash in 93.8%, *tache noire* in 81.8%, arthromyalgia in 21.4%, lymphadenopathy in 6.8% and headache in 26%. In the case series of pediatric patients with MSF, fever was present in 96.1% of patients, rash in 96.1%, *tache noire* in 67.5%, arthromyalgia in 29.6%, lymphadenopathy in 44.2% and headache in 18.3%. In the 52 cases of MSF in which *R. conorii* was identified by molecular methods [6,7,10,11,12,14,15,18,25], fever and rash appeared to be very common in almost all the cases (98% and 96%, respectively); *tache noire* in 46% of cases, arthromyalgia in 25%, headache in 19%; lymphadenopathy was described only in 6% of the cases. The comparison between adults and children symptoms is shown in Figure 3.

In the eight cases of ISF, fever and rash were always present, *tache noire* was present in 5/8, arthromyalgia in 2/8 and headache in 2/8, lymphadenopathy was not found; the mean age was higher than MSF: 53 years. In the cases of HGA, fever was always present, arthromyalgia was present in 7/8 of the cases, headache in 4/8 and rash in 2/8, *tache noire* and lymphadenopathy were not described; mean age was 43.4 years. During HGA, atypical pneumonia, neurological manifestations such as nuchal stiffness, and ulceration of the oral mucosa were observed [35,36,37,38]. Of note was a case of illness in a patient that remained seronegative for 6 months, he was therefore misdiagnosed for a long time and treated for different illnesses before anaplasmosis was diagnosed; meanwhile, his general condition worsened with mild splenomegaly, haemorrhoids, osteopenia, hiatal hernia, gastritis and inflammation of the duodenum not present at first presentation. In this case, the patient was also treated for depression before the correct diagnosis was achieved by PCR [36].

6 cases of TIBOLA were described, of these, 4 were children (3 males, and 1 female) and 2 were adults (2 females) [39,40,41,42]. Fever was present in 5/6 of the cases, *tache noire* was present in all the cases, headache in 3/6 and arthromyalgia in 2/6, the rash was never described; mean age among cases of TIBOLA was 23 years. 

The period of the year in which the illness was diagnosed was often not reported. When data about temporal distribution was available it was seen that the largest majority of cases of MSF occur from May to September. In a large study in Sicily, it was noted that a presentation was more likely to be classic MSF with fever, rash and *tache noire* when observed in summer, while in other seasons the presentation was different and atypical with lymphadenopathy and headache more frequently observed [8]. TIBOLA and anaplasmosis were reported in every season [40]. Importation cases were seen in spring and summer. 

The autochthonous case of murine typhus in Calabria is noteworthy. The patient presented with severe neurologic involvement, dyspnea, septic shock and multi-organ failure with recovery after treatment with doxycycline [46]. 

### 3.2. Complications of Infections Reported in Italy

Many complications were reported in MSF due to *R. conorii* and *R. conorii subsp. israelensis* [15,34]. Secondary hemophagocytic lymphohistiocytosis (sHLH), a life-threatening complication, was fully diagnosed in one pediatric case of serologically diagnosed MSF [7]. Heart involvement was reported three times. In detail, in one report, the presentation was a Kawasaki-like syndrome, in another it was described as myocarditis and in the last, it was reported as atrial fibrillation [6,9,12]. Eye involvement, in the course of MSF, was reported three times [28,29,49]. In all three cases, retinal vasculitis and acute visual loss were reported. The deficit was bilateral in two cases and unilateral in one. In two of the three cases in which retinal vasculitis was reported, there was full recovery after antibiotic therapy. In one of these cases with full recovery, the infection was contracted by direct contact with tick’s blood after it was squashed by the patient; in this case, the presentation was as a Perinaud’s syndrome [29]. In the other, a visual field deficit persisted and areas of capillary non-perfusion on OCT (ocular computer tomography) were evident [28]. Hearing was involved as a permanent and bilateral hearing loss after three days of hospitalization for MSF [24]. In this case, bilateral involvement of the mastoid and a barrier alteration involving the cochlea on the right side was documented with MRI suggesting meningogenic relevance. The result, evident on the auditory evoked potential, was a sensorineural hearing loss. In contrast to MSF, complications were described in all reported ISF cases. The life-threatening conditions included diffuse intravascular coagulation, neurological involvement, multi-organ failure, and septic shock. Other complications observed in Italy were rhabdomyolysis, acute kidney injury, reactivation of herpes virus as herpetic esophagitis, and acute respiratory failure [11,25,30]. Twice, neurological involvement as quadriplegia or as loss of consciousness was observed [11,30]. Respiratory involvement as atypical pneumonia was rarely reported in Italy, the same for pleural effusion [30,31,35]. Mild pleural effusion of low severity was reported in a large cohort of patients with MSF in Sicily and associated with atypical pneumonia [31], isolated atypical pneumonia without pleural effusion was reported in the same cohort and in the course of HGA [35]. In Italian literature, death due to MSF has been reported 12 times [11,15,21,31]. ISF appears to be more severe than MSF. All the 8 cases of ISF were associated with severe complications: neurological involvement as dysarthria, dysdiadochokinesis and neck stiffness [34], disseminated intravascular coagulation (DIC), hepatic necrosis and acute kidney injury were also reported [15], multi-organ failure was diagnosed in 5 patients [15,34]. The case that prompted this study was the first in Italy in which a severe pleural effusion was reported in course of rickettsiosis [3]. In 3 patients with ISF, the disease led to coma and 1 patient died [15].

### 3.3. Pleural Effusion

In the literature, 25 papers describing 487 cases of pleural effusion during Rickettsiales infection were reported globally from 1998 to 2020. 477 of these were cases of scrub typhus, 3 of ehrlichiosis, and 7 of rickettsiosis. All but 1 case of scrub typhus were reported in Asia, more specifically in Thailand, India, China, and South Korea [52]. 

The 25 papers described larger cohorts of patients for a total of 2985 patients with rickettsial diseases, and among these patients, 487 (15.97%) suffered from pleural effusion. Only in 4 of these, molecular techniques were performed to achieve the etiological diagnosis [53,54,55]; the diagnosis was made by microscopic observation of morulae within the cytoplasm of polymorph-nucleate-cells only once [56]; in the other 23 case series, the diagnosis was made by serological assays [52,53,55,57,58,59,60,61,62,63,64,65,66,67,68,69,70,71,72,73]. In the last 25 years in Europe, there was only one report of pleural effusion associated with a rickettsial disease. It was the case of serologically confirmed MSF reported in Greece [74]. In an Asian study, pleural effusion was described in patients with murine typhus and scrub typhus [53]. 

In the case series describing patients with pleural effusion, various complications were mostly associated to scrub typhus, as adult respiratory distress syndrome, sepsis with multi-organ failure, acute kidney injury, meningitis, DIC or severe thrombocytopenia were very frequently reported. 59 of the above 487 patients with pleural effusion died [53,59,60,61,65,66,69,75].

## 4. Discussion

The many reported rickettsioses in Italy are MSF, TIBOLA and HGA. MSF in Italy may be caused by *R. conorii conorii*, *R. conorii subsp. indica*, *R. conorii subsp. israelensis*, *R. massiliae*, *R. monacensis* and *R. slovaca*. The clinical picture of MSF caused by *R. conorii subsp. israelensis* should be more correctly named ISF. MSF and ISF may be associated with complications mainly due to systemic vasculitis, and to the severe inflammatory response to the infection [76] MSF and ISF can trigger the development of sHLH, a condition associated with an intense release of cytokines [77]. Other vasculitic manifestations different to ophthalmic vein thrombosis were reported. These were hepatitis, hepatic failure, acute kidney injury, neurosensory hearing loss and neurological involvement.

ISF is a disease similar to MSF but characterized by higher severity. The *tache noire* in ISF was classically considered absent according to the first descriptions [78]. Conversely, De Sousa et al. in Portugal reported a cohort in which the eschar was present in 17/45 patients [79]. Similarly, *tache noire* was present in 5/8 of the cases of ISF reported in Italy. In the study of De Sousa, gastrointestinal symptoms were a predictor of poor outcome; however, the pathophysiology of the gastrointestinal symptoms remained unclear. The study suggested that vomiting, nausea and diarrhoea could be related to the rise in intracranial pressure and, therefore, to neurological involvement, or a consequence of the massive release of inflammatory cytokines. In ISF the injury to vascular endothelium is multi-systemic and more severe than in MSF. The manifestations can include confusion, for the involvement of the brain endothelium, tachypnea, and atypical pneumonia (for the involvement of the lung endothelium and the sepsis status), and petechial exanthema (for the involvement of skin endothelium, thrombocytopenia, and alterations of blood coagulation) [79]. Also, acute kidney injury due to sepsis and systemic hypoperfusion can occur. Some cases of purpura fulminans associated with ISF were reported in Israel [80,81]. *R. conorii subsp. israelensis* was first reported in Italy in 2005, in fact, it was identified in blood samples of patients with MSF collected from 1987 and 2001 [15]. Other countries in which the microorganism is known to be present are Portugal, Israel, and other nearby countries between the Maghreb and the Middle East [34]. The reported imported cases also included one notified in the United Kingdom, in a traveler who returned from south Portugal and died in 2005 [82], and another who died in Switzerland in 2006 after a cruise in the south Mediterranean Sea [83]

SENLAT (scalp eschar and neck lymphadenopathy after tick bite), the acronym was suggested by the Marseille group to summarize the features of the illness without a precise microbiological association, so diseases related with *R. rioja, R. raoultii, R. slovaca, R. massiliae, Coxiella burnetii, Bartonella henselae, Borrelia burgdoferi* and other tick-borne pathogens can be clinically unified [84]. Some cases of clinical diagnosis of TIBOLA due to *R. slovaka* were reported in Italy. The diagnosis in these cases were achieved tacking in account information about the ticks (*Dermacentor*), clinical manifestations and full recovery after treatment with doxycycline [39]. Other names suggested for the illness are the less specific TIBOLA (tick-borne lymphadenopathy) and the more restrictive DEBONEL (Dermacentor borne necrosis eschar and lymphadenopathy) [84]. *Dermacentor* spp. is active prevalently in winter and fall in contrast to *Rhipicephalus* spp. It waits for its hosts in vegetation, usually 1–1.5 m from the soil and prefers hairy animals, indeed it has been found frequently in mountainous territories and feeding on wild boars. As the association with the scalp as the site of the bite appears natural, so does the lymphadenopathy of the neck [40]. SENLAT is more present in children and women. *R. massiliae* and *R. slovaca* were identified in Italy by PCR in unwell patients with TIBOLA [41,42]. The other rickettsia species associated with TIBOLA are present in Italy, even if never identified in Italian human cases of TIBOLA [85]. 

All the Italian cases of rickettsiosis caused by *R. africae* occurred in travelers from Africa [43,44,48]; they presented with a clinical picture of ATBF [43,44] and a case of sacral syndrome [48]. Only one case of infection by *R. aeschlimannii* was reported in Italy; it was an autochthonous case that occurred in a man with fever and arthralgia and a sharp rise in serum hepatic enzyme [47]. In this case, the whole blood was negative to all tests to identify *Rickettsia* and the diagnosis was achieved by liver biopsy. In the past, both *R. africae* and *R. aeschlimannii* were known as African microorganisms, however, the route of migratory birds has brought infected vectors in Italy and climatic changes permit their survival during cold seasons [86]. In the past years, the colonization of territories outside Africa by the vectors for *R. africae* and *R. aeschlimannii* due to the raising of the mean temperature was theorized. In Italy, the territory seen as more vulnerable were the major islands; indeed, ticks belonging to *Hyalomma* and *Amblyomma* and the microorganism typically associated with them were found different times [85,86,87]. Furthermore, rickettsioses follow the vector activities seasonally and in geographical distribution, in recent years the change of mean temperature influenced the behaviors of the vector. In United State, recently a raising in the incidence of Lyme disease had been described [88]; it could be due to the changes in the activity of the arthropods and it could be associated with climate change; regarding rickettsial disease, modification of incidence could be observed in next years; to date, a raising in the number of species of *Rickettsia* and their vectors is reported [85]. MSF and ISF are mostly associated with *Rhipicephalus sanguineus* and other ticks active in summer; TIBOLA is associated with *Dermacentor* ticks, more common in cold areas and cold seasons, so this illness has no seasonal preference [40]. The previous observation of different presentations between MSF in summer and winter could be due to different species of *Rickettsia,* misdiagnosed because of cross-reaction between anti-*Rickettsia* antibodies through the species [8]. Indeed, in the past, before molecular investigations were available, all cases of MSF were attributed to *R. conorii.* Of note, in a large study on ticks collected from humans, it was found that in Sicily *R. massiliae* was far more common than *R. conorii* despite the lower number of confirmed cases for the first [89]. 

Human granulocytic anaplasmosis caused by *A. phagocytophilum* in Italy were diagnosed 7 times [35,36,37,38]; 2 cases of anaplasmosis without identification of the species were further reported [18]. Fever was present in all of the cases; other non-specific symptoms were variably reported. HGA may have a subacute presentation, fever, sometimes rash and a slow clinical course [90] So, the diagnosis could be missed while the patient’s general condition slowly worsens. The case report of a Sicilian man misdiagnosed for 6 months and treated also for depression is a clear example of this [36]. Since the first identification of a case of HGA in Slovenia in 1997, and a positive human sample in 1995, the illness became not uncommon across Europe. Indeed, a serological survey shows that human infection could be underreported, and a good number of patients could be asymptomatic or recover without a diagnosis of anaplasmosis [90,91]. 

Ehrlichiosis was described only once in Italy. In this sole case, the diagnosis was made only with a serological assay, hence the possibility of a cross-reaction, which cannot be excluded. Murine typhus, endemic in Italy before 1950, has only been reported once in the last 25 years [1,46]. 

According to the Italian Health Ministry, mortality in rickettsiosis in Italy is about 3%. Nonetheless, in our series, the death occurred only 13/2831 (0.46%) of the cases, a much lower figure [1]. Based on our experience, fatal cases have often been observed in small-town hospitals and these cases have hardly been the subject of publication.

Scrub typhus in Italy in the last 25 years was only once reported in a study has describing a group of three Italian travelers returning from Laos. All three patients recovered after antibiotic therapy. Endemic in the far east, *O. tsutsugamushi* is the etiological agent of scrub typhus, an illness characterized by headache, fever, muscle pain, lymphadenopathy, maculopapular rash and eschar at the site of the bite of the vector, which is usually a chigger [92]. In Italy, *O. tsutsugamushi* was never identified in vectors nor autochthonous cases of scrub typhus were described.

The species of Rickettsiales more commonly involved in pleurisy and atypical pneumonia was *O. tsutsugamushi.* Cases of scrub typhus with pleural effusion were mostly described in the Far East. Imported cases were also described in German tourists returning from endemic areas [52,93,94]. The largest number of cases of pleural effusion came from South Korea, China and India. One other Rickettsiales identified in patients with pleural effusion was *E. chaffeensis* [54,75]. In one of the above cases of HME, *Ehrlichia* was identified by microscopy [56]. Furthermore, in the study of Le Van et al., many cases of pleural involvement or pleural effusion were associated with murine typhus [53]. Pleurisy associated with *R. conorii* or *R. conorii subsp. israelensis* infection is an extremely unusual clinical manifestation and has been reported only twice in the last 25 years [3,74,95,96,97,98].

## 5. Conclusions

MSF, TIBOLA and HGA are the main human rickettsial diseases present in Italy. MSF, especially when caused by *R. conorii subsp. israelensis* (also known as Israeli Spotted Fever)*,* may be a severe disease. A high index of suspicion is required to promptly start life-saving therapy. Pleural effusion and interstitial pneumonia may be part of the clinical picture of severe rickettsial disease and should not lead the physician away from this diagnosis.

## Figures and Tables

**Table 1:** Demographic and clinical features of patients with diagnosis of MSF.

Number of Patients	2785
Adults (>18y)	192
Children	1204
Age not reported	1389
**Mean age**	12.3 years
Mean age of adults	47.7 years
Mean age of children	5.9 years
Male	856
Female	540
Male/Female ratio	1.6
Sex not reported	1399
**Symptoms**	
Fever	96.1%
Rash	95.8%
*Tache noire*	70.0%
Arthromyalgia	31.6%
Lymphadenopathy	39.4%
Headache	22.3%

## Supplementary Materials

The following supporting information can be downloaded at: https://www.mdpi.com/article/10.3390/tropicalmed7010011/s1. The Supplementary File containing 3 Excel sheets: (1) Rickettsiales infection in Italy; (2) atypical presentation in Italy and (3) pleural effusion in course of infection by Rickettsiales in the world.
